# *De novo* transcriptome assemblies of five major European oilseed rape insect pests

**DOI:** 10.1186/s12863-023-01115-8

**Published:** 2023-03-10

**Authors:** Salma Sana, Ines Vollhardt, Katharina Kubon, Michael Rostás, Stefan Scholten

**Affiliations:** 1 Division of Crop Plant Genetics, Department of Crop Science, Georg-August-University Goettingen, Goettingen, Germany; 2 Division of Agricultural Entomology, Department of Crop Science, Georg-August-University Goettingen, Goettingen, Germany; 3 Center for integrated Breeding Research (CiBreed), Goettingen, Germany

**Keywords:** Transcriptome, RNA-seq, Trinity assembly, Insect pests, Oilseed rape

## Abstract

**Objective:**

Insect pests can cause severe losses in oilseed rape yields across Europe. Genomic and transcriptomic information is very limited for these insects. The aim of our study was to provide transcriptomic resources on several oilseed rape herbivores that will support research into their biology and help develop new methods of sustainable pest management.

**Data:**

Transcriptomes for larval stages of five major European pest species were *de novo* assembled by Trinity assembler. Total number of transcripts ranged from 112,247 for *Ceutorhynchus pallidactylus* to 225,110 for *Ceutorhyncus napi.* Intermediate numbers of 140,588, 140,998 and 144,504, were found for *Psylliodes chrysocephala*, *Dasineura brassicae, and Brassicogethes aeneus*, respectively. Bench-marking universal single-copy orthologues analyses for each dataset indicated high degree of completeness for all five species. The transcriptomes extend the list of genomic data on insect larvae that constitute major pests of oilseed rape. The data provide information on larval physiology and form a basis to develop highly specific RNA interference-based plant protection.

## Objective

*Brassica napus* (oilseed rape, OSR) in the mustard family Brassicaceae is the second most important oilseed crop. However, since 1990 the average yields of OSR have been declining in Europe and Australia [1]. These declines have not mainly been linked to an increase in pests and pathogens but also to warm temperatures and low precipitation [2]. In Europe, the ban on the use of three neonicotinoids as active ingredients for seed treatment has also exacerbated pest problems [3]. Overall, insects account for an average yield loss of 13% on the global and 15% on a European scale [1].

Cabbage stem weevil *Ceutorhynchus pallidactylus* (Coleoptera, Curculionidae), rape stem weevil *Ceutorhynchus napi* (Coleoptera, Curculionidae), pollen beetle *Brassicogethes aeneus* (Coleoptera, Nitidulidae), cabbage stem flea beetle *Psylliodes chrysocephala* (Coleoptera, Chrysomelidae) and Brassica pod midge *Dasineura brassicae* (Diptera, Cecidomyiidae) are the most important insect pests affecting European oilseed rape crops [1]. These insects generally affect OSR in the larval stage although significant damage can also be caused by adult *B. aeneus*, which migrates into OSR field to feed on pollen and buds or adult *P. chrysocephala* that feed on seedlings [4]. The larvae of the weevils *C. pallidactylus* and *C. napi* feed inside the OSR stem, resulting in fewer pod production and severe losses [5], whereas larvae of *P. chrysocephala* mine the petiole [6]. The larvae of the midge *D. brassicae* feed on the inner pod wall and cause a complete loss of seeds [7].

Basic genomic information is lacking for these pest species except for *B. aeneus* (GCA_921294245.1- which is genomic assembly data) and GAPE01000000 a transcriptomic data set obtained from adult stage *B. aeneus* [8]. In this study, we have sequenced, assembled and annotated transcriptome assemblies for the larvae of the above-mentioned pests. This will lay the foundation for genomic and transcriptomic resources for these pest species, which can be utilized to develop methods of pest control in *Brassica napus*, such as host-induced gene silencing (HIGS) technology and spray-induced gene silencing (SIGS). These techniques have enormous potential as an eco-friendly alternative to pesticides for crop protection [9]. Additionally, these transcriptome resources will also be helpful to better understand the physiology and biochemistry of these insect species.

## Data description

The larvae of *C. napi* were collected from infested summer oilseed rape after rearing the larvae for one generation. To infect the plants, beetles that were hatched in the lab from larvae collected in an experimental winter oilseed rape field in Lower Saxony, Germany in 2019 were used. Larvae of all other species were used directly for transcriptome analysis after collection. *C. pallidactylus Msh*. larvae were collected from the main shoots of plants from an experimental winter oilseed rape in Lower Saxony, Germany in 2020. Samples of *P. chrysocephala* (L.) were isolated from the petioles of the leaves of plants from an experimental winter oilseed rape field in Mecklenburg-Western Pomerania, Germany in 2020. The larvae of *B. aeneus* (F.) and *D. brassicae* (Macquart) were collected from the flowers and pods respectively of winter oilseed rape plants in an experimental field from the University of Göttingen in Göttingen in 2020. Based on macroscopic morphological characters, we sampled mixed first- to third-instar larvae of *C. pallidactylus*, *B. aeneus*, and *D. brassicae*, while *C. napi* sampling was biased to the late second- to early third-instar and *P. chrysocephala* sampling was biased to the first- to early second-instar.

## RNA isolation and Illumina sequencing

Total RNA was extracted with Quick-RNA Tissue/Insect Kit (Zymo Research, Freiburg, Germany) according to manufacturer’s instructions. Stranded libraries were prepared from 1 µg total RNA after poly A-based mRNA isolation with NEBNext Ultra II Directional RNA library preparation kit (NEB, Frankfurt am Main, Germany) following the manufacturer’s guidelines.

2 × 100 bp paired-end read sequencing by DNA nanoball sequencing technology (DNBSEQ) was performed at BGI Genomics, Hong Kong.

## Data filtering, transcriptome assembly and quality

The raw reads were assessed for quality by FASTQC and cut adapt was used to remove adaptor sequences [10]. *De novo* assemblies of the transcriptomes of five samples were performed from the RNA-seq data. The transcriptomes assemblies were generated with Trinity software version (2.11.0) [11] with default settings. The transcriptome assemblies were filtered for *Brassica napus* transcripts prior to submission to GenBank. Transvestigator [12] was implemented to prepare the transcriptome assemblies for the submission which ensures that the predicted ORFs are in positive strand (Data file 1). The associated raw data for each species are presented in Data files 2 to 6. The total number of transcripts was 112,247 for *C. pallidactylus*, 140,588 for *P. chrysocephala*, 140,998 for *D. brassicae*, 144,504 for *B. aeneus*, and 225,110 for *C. napi.* Based on all transcript contigs, the average contig length was highest for *C. pallidactylus* with 1543 bp and lowest for *C. napi* with 936.03 bp. Intermediate average contig lengths were found for *D. brassicae, P. chrysocephala, and B. aeneus* with 1476.4, 1461.07, and 1135.3 bp, respectively. Detailed assembly statistics for all samples are summarized in Data file 7. The completeness of the transcriptome assemblies was determined by BUSCO (Benchmarking Universal Single-Copy Orthologs) [13]. BUSCO searches against 3285 marker genes from diptera lineage found 84.1%, of complete universal single-copy genes for *D. brassicae*; BUSCO searches against 2124 marker genes from Endopterygota lineage found completeness of 95.4% for *C. pallidactylus* and *B. aeneus*, 94.3% for *C. napi*, and 92.9% for *P. chrysocephala*, which indicates a moderate to high level of completeness for all species (Data file 8).

## Annotation

Functional annotation of the transcriptome assemblies generated by Trinity was performed by Trinotate v3.2.2 pipeline (https://trinotate.github.io/) [14]. The trinotate software automates the functional annotation of the transcripts and predicted protein sequences. The Trinotate pipeline employs several software including Hmmer v.3.1b1 [15], a protein domain identification (PFAM) software [16], Tmhmm v.2.0c prediction of transmembrane helices in proteins [17], Rnammer v.1.2 to predict ribosomal RNA [18], SignalP v.4.1 predicts signal peptide cleavage sites [19], prediction of gene ontology GOseq [20], eggnog v.3.0 search for orthologous groups [21]. Open reading frames (ORFs) were predicted with Transdecoder v5.5.0 (http://transdecoder.github.io) and only those ORFs were retained that had a minimum length of at least 100 amino acids, which were then blasted using BlastP against NCBI protein database with an E-value cut-off of 10 − 3 [22]. Finally, all the hits were loaded into a Sqlite database generated by Trinotate to produce a full annotation report for each species (Data file 9).

For the efficiency of HIGS and SIGS technology, the uptake and systemic transport of double stranded RNA (dsRNA) in insect pests is important [23]. One pathway possibly involved in these processes is mediated by *Sid-1-like (SIL)* genes, encoding dsRNA-selective, dsRNA-gated channel proteins [23]. Because the number of *SIL* genes are highly variable between insect species [24], we searched for expressed orthologs and found expression of *SIL* gene orthologues in larvae of three species: Three annotated orthologues were found in *C. napi* and two each in *C. pallidactylus* and *B. aeneus* (Data file 9).

Beside the uptake and systemic transport of dsRNA in insects, the development of species-specific dsRNAs for RNA interference against essential target genes is required for HIGS and SIGS technology [9]. Previously, *Sect. 23*, a subunit of the coat protein complex II vesicle transport complex that promote transport vesicle formation in the endoplasmatic reticulum was proven to be an effective target gene leading to high mortality in Colorado potato beetle, *Leptinotarsa decemlineata* [25] and western corn rootworm, *Diabrotica virgifera virgifera* [26]. In larvae of all five species analyzed, transcripts of orthologues of *Sect. 23* were identified (Data file 9). Given their previous effectivity, these transcript sequences might provide a starting point to test the susceptibility of the five OSR pests for dsRNA-mediated gene silencing and develop species-specific HIGS and SIGS approaches.

**Table 1:** Overview of data files/data sets

Label	Name of data file/data set	File types (file extension)	Data repository and identifier (DOI or accession number)
*Data file 1*	*Transcriptome assemblies of five insect species*	SRA, TSA files (.fastq, .fasta)	NCBI *BioProject*https://identifiers.org/bioproject:PRJNA807498 [27]
*Data file 2*	*RNA-seq* of *Ceutorhynchus pallidactylus*	SRA file (.fastq)	NCBI *Sequence Read Archive*https://identifiers.org/insdc.sra:SRR18053996 [28]
*Data file 3*	*RNA-seq* of *Ceutorhynchus napi*	SRA file (.fastq)	NCBI *Sequence Read Archive*https://identifiers.org/insdc.sra:SRR18053995 [29]
*Data file 4*	*RNA-seq* of *Psylliodes chrysocephala*	SRA file (.fastq)	NCBI *Sequence Read Archive*https://identifiers.org/insdc.sra:SRR18053993 [30]
*Data file 5*	*RNA-seq* of *Brassicogethes aeneus*	SRA file (.fastq)	NCBI *Sequence Read Archive*https://identifiers.org/insdc.sra:SRR18053994 [31]
*Data file 6*	*RNA-seq* of *Dasineura brassicae*	SRA file (.fastq)	NCBI *Sequence Read Archive*https://identifiers.org/insdc.sra:SRR18053992 [32]
*Data file 7*	*Table 1* Transcriptome assembly statistics for larvae of Brassica napus pest insects	Document file (.docx)	Figshare, 10.6084/m9.figshare.19705516.v2 [33]
*Data file 8*	*Table 2* Benchmarking Universal Single-Copy Orthologues (BUSCO) analysis on transcriptome assemblies of larvae of Brassica napus pest insects	Document file (.docx)	Figshare, 10.6084/m9.figshare.19705513.v1 [34]
*Data file 9*	*Annotation reports*	Speadsheet (.xlsx)	Figshare, 10.6084/m9.figshare.21758303 [35]

## Limitations

The insect larvae pooled were not staged i.e. they do not have specifically defined developmental stages. Additionally, no biological replicates were performed.

## Data Availability

The transcriptome assemblies have been deposited in the NCBI BioProject PRJNA807498 (https://identifiers.org/bioproject:PRJNA807498) with raw data separately (https://identifiers.org/insdc.sra:SRR18053996, https://identifiers.org/insdc.sra:SRR18053995, https://identifiers.org/insdc.sra:SRR18053993, https://identifiers.org/insdc.sra:SRR18053994, and https://identifiers.org/insdc.sra:SRR18053992). Please see Table 1 for details and references [33–35] for the final products of analyses submitted to *figshare* for public usage.

## References

[1] Zheng X, Koopmann B, Ulber B, von Tiedemann A. A Global Survey on Diseases and Pests in Oilseed Rape—Current Challenges and Innovative Strategies of Control.Frontiers in Agronomy. 2020;2.

[2] Assefa Y, Prasad PVV, Foster C, Wright Y, Young S, Bradley P (2018). Major management factors determining Spring and Winter Canola Yield in North America. Crop Sci.

[3] Lundin O (2021). Consequences of the neonicotinoid seed treatment ban on oilseed rape production – what can be learnt from the swedish experience?. Pest Manag Sci.

[4] Juhel AS, Barbu CM, Franck P, Roger-Estrade J, Butier A, Bazot M (2017). Characterization of the pollen beetle, Brassicogethes aeneus, dispersal from woodlands to winter oilseed rape fields. PLoS ONE.

[5] Milovac Ž, Zorić M, Franeta F, Terzić S, Petrović Obradović O, Marjanović Jeromela A (2017). Analysis of oilseed rape stem weevil chemical control using a damage rating scale. Pest Manag Sci.

[6] Stará J, Kocourek F (2019). Cabbage stem flea beetle’s (Psylliodes chrysocephala L.) susceptibility to pyrethroids and tolerance to thiacloprid in the Czech Republic. PLoS ONE.

[7] Murchie AK, Hume KD (2003). Evidence for monogeny in the brassica pod midge Dasineura brassicae. Entomol Exp Appl.

[8] Zimmer CT, Maiwald F, Schorn C, Bass C, Ott M-C, Nauen R (2014). A de novo transcriptome of european pollen beetle populations and its analysis, with special reference to insecticide action and resistance. Insect Mol Biol.

[9] Sang H, Kim J-I (2020). Advanced strategies to control plant pathogenic fungi by host-induced gene silencing (HIGS) and spray-induced gene silencing (SIGS). Plant Biotechnol Rep.

[10] Martin M (2011). Cutadapt removes adapter sequences from high-throughput sequencing reads. EMBnet J.

[11] Grabherr MG, Haas BJ, Yassour M, Levin JZ, Thompson DA, Amit I (2011). Full-length transcriptome assembly from RNA-Seq data without a reference genome. Nat Biotechnol.

[12] DeRego T, Hall B. ben-guin, Geib S. Transvestigator early release. 2014.

[13] Simão FA, Waterhouse RM, Ioannidis P, Kriventseva EV, Zdobnov EM (2015). BUSCO: assessing genome assembly and annotation completeness with single-copy orthologs. Bioinformatics.

[14] Bryant DM, Johnson K, DiTommaso T, Tickle T, Couger MB, Payzin-Dogru D (2017). A tissue-mapped Axolotl De Novo Transcriptome enables identification of limb regeneration factors. Cell Rep.

[15] Finn RD, Clements J, Eddy SR (2011). HMMER web server: interactive sequence similarity searching. Nucleic Acids Res.

[16] Finn RD, Bateman A, Clements J, Coggill P, Eberhardt RY, Eddy SR (2014). Pfam: the protein families database. Nucleic Acids Res.

[17] Krogh A, Larsson B, von Heijne G, Sonnhammer EL (2001). Predicting transmembrane protein topology with a hidden Markov model: application to complete genomes. J Mol Biol.

[18] Lagesen K, Hallin P, Rødland EA, Staerfeldt H-H, Rognes T, Ussery DW (2007). RNAmmer: consistent and rapid annotation of ribosomal RNA genes. Nucleic Acids Res.

[19] Petersen TN, Brunak S, von Heijne G, Nielsen H (2011). SignalP 4.0: discriminating signal peptides from transmembrane regions. Nat Methods.

[20] Young MD, Wakefield MJ, Smyth GK, Oshlack A (2010). Gene ontology analysis for RNA-seq: accounting for selection bias. Genome Biol.

[21] Powell S, Szklarczyk D, Trachana K, Roth A, Kuhn M, Muller J et al. eggNOG v3.0: orthologous groups covering 1133 organisms at 41 different taxonomic ranges.Nucleic Acids Res. 2012;40 Database issue:D284–289. 10.1093/nar/gkr1060 PMC3245133 22096231

[22] Altschul SF, Gish W, Miller W, Myers EW, Lipman DJ (1990). Basic local alignment search tool. J Mol Biol.

[23] Liu S, Jaouannet M, Dempsey DA, Imani J, Coustau C, Kogel K-H (2020). RNA-based technologies for insect control in plant production. Biotechnol Adv.

[24] Joga MR, Zotti MJ, Smagghe G, Christiaens O, RNAi (2016). Efficiency, systemic Properties, and Novel Delivery Methods for Pest Insect Control: what we know so far. Front Physiol.

[25] Zhu F, Xu J, Palli R, Ferguson J, Palli SR (2011). Ingested RNA interference for managing the populations of the Colorado potato beetle, Leptinotarsa decemlineata. Pest Manag Sci.

[26] Vélez AM, Fishilevich E, Rangasamy M, Khajuria C, McCaskill DG, Pereira AE (2020). Control of western corn rootworm via RNAi traits in maize: lethal and sublethal effects of Sect. 23 dsRNA. Pest Manag Sci.

[27] Sana S, Vollhardt I, Kubon K, Rostás M, Scholten S. *De novo* transcriptome assemblies of five major European oilseed rape insect pests.BioProject. 2022. https://identifiers.org/bioproject:PRJNA807498. 10.1186/s12863-023-01115-8 PMC10007812 36899327

[28] Sana S, Vollhardt I, Kubon K, Rostás M, Scholten S. *De novo* transcriptome assemblies of five major European oilseed rape insect pests.Sequence Read Archive. 2022. https://identifiers.org/insdc.sra:SRR18053996. 10.1186/s12863-023-01115-8 PMC10007812 36899327

[29] Sana S, Vollhardt I, Kubon K, Rostás M, Scholten S. *De novo* transcriptome assemblies of five major European oilseed rape insect pests.Sequence Read Archive. 2022. https://identifiers.org/insdc.sra:SRR18053995. 10.1186/s12863-023-01115-8 PMC10007812 36899327

[30] Sana S, Vollhardt I, Kubon K, Rostás M, Scholten S. *De novo* transcriptome assemblies of five major European oilseed rape insect pests.Sequence Read Archive. 2022. https://identifiers.org/insdc.sra:SRR18053993. 10.1186/s12863-023-01115-8 PMC10007812 36899327

[31] Sana S, Vollhardt I, Kubon K, Rostás M, Scholten S. *De novo* transcriptome assemblies of five major European oilseed rape insect pests.Sequence Read Archive. 2022. https://identifiers.org/insdc.sra:SRR18053994. 10.1186/s12863-023-01115-8 PMC10007812 36899327

[32] Sana S, Vollhardt I, Kubon K, Rostás M, Scholten S. *De novo* transcriptome assemblies of five major European oilseed rape insect pests.Sequence Read Archive. 2022. https://identifiers.org/insdc.sra:SRR18053992. 10.1186/s12863-023-01115-8 PMC10007812 36899327

[33] Sana S, Vollhardt I, Kubon K, Rostás M, Scholten S. *De novo* transcriptome assemblies of five major European oilseed rape insect pests. Figshare. 2022 10.6084/m9.figshare.19705516.v2. 10.1186/s12863-023-01115-8 PMC10007812 36899327

[34] Sana S, Vollhardt I, Kubon K, Rostás M, Scholten S. *De novo* transcriptome assemblies of five major European oilseed rape insect pests. Figshare. 2022. 10.6084/m9.figshare.19705513.v1. 10.1186/s12863-023-01115-8 PMC10007812 36899327

[35] Sana S, Vollhardt I, Kubon K, Rostás M, Scholten S. *De novo* transcriptome assemblies of five major European oilseed rape insect pests. Figshare. 2022. 10.6084/m9.figshare.21758303. 10.1186/s12863-023-01115-8 PMC10007812 36899327

